# Investigating a *Plasmodium falciparum* erythrocyte invasion phenotype switch at the whole transcriptome level

**DOI:** 10.1038/s41598-019-56386-y

**Published:** 2020-01-14

**Authors:** Prince B. Nyarko, Sarah J. Tarr, Yaw Aniweh, Lindsay B. Stewart, David J. Conway, Gordon A. Awandare

**Affiliations:** 1 West African Centre for Cell Biology of Infectious Pathogens, University of Ghana, Legon, Accra, Ghana; 2 Department of Biochemistry, Cell and Molecular Biology, University of Ghana, Legon, Accra, Ghana; 3 Department of Pathogen Molecular Biology, London School of Hygiene and Tropical Medicine, London, WC1E 7HT United Kingdom

**Keywords:** Transcriptomics, Malaria

## Abstract

The central role that erythrocyte invasion plays in *Plasmodium falciparum* survival and reproduction makes this process an attractive target for therapeutic or vaccine development. However, multiple invasion-related genes with complementary and overlapping functions afford the parasite the plasticity to vary ligands used for invasion, leading to phenotypic variation and immune evasion. Overcoming the challenge posed by redundant ligands requires a deeper understanding of conditions that select for variant phenotypes and the molecular mediators. While host factors including receptor heterogeneity and acquired immune responses may drive parasite phenotypic variation, we have previously shown that host-independent changes in invasion phenotype can be achieved by continuous culturing of the W2mef and Dd2 *P. falciparum* strains in moving suspension as opposed to static conditions. Here, we have used a highly biologically replicated whole transcriptome sequencing approach to identify the molecular signatures of variation associated with the phenotype switch. The data show increased expression of particular invasion-related genes in switched parasites, as well as a large number of genes encoding proteins that are either exported or form part of the export machinery. The genes with most markedly increased expression included members of the erythrocyte binding antigens (*EBA*), reticulocyte binding homologues (*RH*), surface associated interspersed proteins (*SURFIN*), exported protein family 1 (*EPF1*) and Plasmodium Helical Interspersed Sub-Telomeric (*PHIST*) gene families. The data indicate changes in expression of a repertoire of genes not previously associated with erythrocyte invasion phenotypes, suggesting the possibility that moving suspension culture may also select for other traits.

## Introduction

*Plasmodium falciparum* malaria remains a major global public health challenge^1–3^. The 48-hour cyclical asexual replication of the blood stage parasite is responsible for the clinical symptoms of the infection^4^. Parasite control is hampered by genetic and phenotypic variations that impact negatively on drug and vaccine development strategies. Thus, a better understanding of the molecular mechanisms responsible for parasite phenotypic variation is important for the development and application of new malaria control strategies.

Erythrocyte invasion by *P. falciparum* merozoites has been a subject of significant research interest due to its central role in parasite survival and transmission^5–7^. Some of these studies have demonstrated the importance of invasion-related gene families in the parasite genome, particularly the erythrocyte binding antigens (EBAs) and reticulocyte binding-like homologues (RHs)^5,8–16^. The array of different genes involved in invasion allows the parasite to vary ligands used for invasion^17–20^, enabling adaptation to differences in host environments including erythrocyte receptor heterogeneity and ligand-specific immune responses^21–25^. Expression and usage of particular ligands appear to depend on the genetic background of the parasite strain^11,16,18,26–28^, with some strains requiring sialic acids on the erythrocyte surface for effective invasion while others do not^10,17,29–33^.

Some of the sialic acid-dependent strains (particularly W2mef, Dd2 and CSL2) have the propensity to switch invasion phenotype when selected on sialic acid-deficient erythrocytes^18,20,34^. Recently, we discovered another remarkable feature of W2mef and Dd2. When continuously cultivated in moving suspension cultures, these strains gradually gain the ability to invade neuraminidase-treated (sialic acid-deficient) erythrocytes, but remain sialic acid-dependent when kept in continuous static cultures^17^. This observation was surprising given that no modifications were made to either the parasites or erythrocytes used for culturing. Interestingly, the observed phenotype selected by moving suspension culture was similar to that achieved in other experiments by selection on sialic acid-deficient erythrocytes, and similarly associated with greatly increased expression of the *Rh4* and *eba165* genes. We considered the possibility that the phenotypic switch to sialic acid-independent invasion may also involve genes that are not yet known to be associated with different invasion pathways. To this end, we conducted whole transcriptome analysis of schizonts from parasite cultures that had switched invasion pathways during suspension culture, compared with schizonts from unswitched static cultures, using highly biologically replicated samples^35^. This has revealed the differential expression of a larger repertoire of genes likely to be involved in invasion phenotype switching, including known invasion ligands as well as gene families not previously implicated in erythrocyte invasion.

## Results

### Sequencing of biological replicates and identification of differentially expressed genes

**Figure 1:**
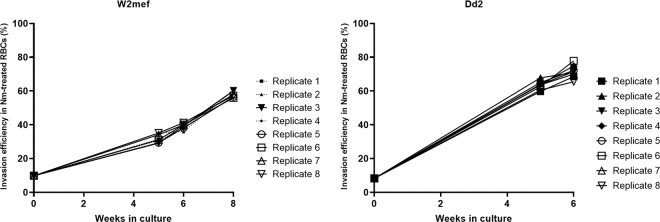
Erythrocyte invasion history of parasite cultures used for primary analyses. Efficiency of invasion into neuraminidase treated erythrocytes by all biological replicates of static cultured baseline parasites (week 0) and their respective suspended cultures were monitored at regular intervals by invasion assays. Baseline cultures were confirmed to be SA-dependent, while suspended parasites gradually acquired SA-independent invasion phenotype with increasing length of time in culture. Schizonts were harvested at baseline, and weeks 8 and 6, respectively for suspended W2mef (**a**) and Dd2 (**b**). Broken lines indicate suspended replicates which were not sequenced.

Parasite schizont samples were generated for baseline (*Baseline*; BL), moving suspension (*Suspended*; SP) and static control (*Static*; ST) cultures of *P. falciparum* W2mef and Dd2 strains, using eight replicate cultures of each condition. The switched invasion phenotypes of all SP culture replicates were confirmed, with invasion assays showing that they had acquired the ability to invade neuraminidase-treated erythrocytes (Fig. 1), while ST cultures remained similar to BL. Schizonts were harvested from each culture replicate and total RNA extracted for sequencing. Quality controls were performed at the RNA extraction, library preparation and sequencing steps. After quality control filtering, whole transcriptome data for analysis were obtained for eight replicates for each of the W2mef and Dd2 baseline cultures (W2mef BL and Dd2 BL, respectively), eight replicates of the Dd2 suspended cultures (Dd2 SP), five replicates of the W2mef suspended cultures (W2mef SP), and two replicates of each of the W2mef and Dd2 static control cultures (W2mef ST and Dd2 ST, respectively), and analysed using the DESeq2 R package^36^. Samples that could not be sequenced had insufficient RNA (≥500 ng) for library preparation.

Reads were uniquely mapped to a total of 5072 *P. falciparum* genes (Supplementary Tables S1 and S2), with very wide variation in mapping depth among the genes as expected (transformed log2 FPKM ranging from 15.42 to 0.16; Supplementary Table S3). Principal component analysis (PCA) showed overlaps among members of sample groups, with no specific group clustering (Supplementary Fig. S1). This was expected since we envisioned only subtle differences to exist between suspended and static cultured parasites. Nonetheless, to test the robustness of our approach, we checked for outliers using default parameters of the DaMiRseq package^37^ and removed samples with mean absolute correlation ≤0.85. We then performed differential expression analysis with DESeq2 using retained samples while correcting for any batch effects (designated here as corrected analysis). A correlation of the fold change values for corrected vs uncorrected analysis shows a near perfect linear relationship, with a correlation coefficient of 0.96 (Supplementary Fig. S2). Furthermore, we correlated the FPKM values of all samples to the FPKMs of data from a previous time-course study^38^ and confirmed that all samples had peak expression at 40–48 hours post invasion (Supplementary Fig. S3). This was considered as a validation of well-synchronized cultures and therefore, qualifying all samples for inclusion in the subsequent analyses. Based on the results of our aforementioned quality checks we resolved to include all samples in our primary analysis.

To limit statistical noise and false discovery, we first compared the ST cultures to BL cultures to determine the extent of any background noise generated from culturing in general. We observed no significantly differentially expressed genes between ST and BL cultures in either strain when an adjusted *P* value < 0.1 was applied as the level of significance (Supplementary Tables S4 and S5). This was as expected, since ST cultures were phenotypically similar to BL cultures. This observation also allowed us to confidently use the highly replicated BL cultures as an alternative to ST cultures as comparator against SP cultures in the differential expression analysis. A more stringent adjusted *P* value (<0.01)^35^ was however applied for the transcriptome screen to identify significantly differentially expressed genes in the SP cultures relative to BL cultures.

### Genes differentially expressed between static and moving suspension cultures of W2mef

**Figure 2:**
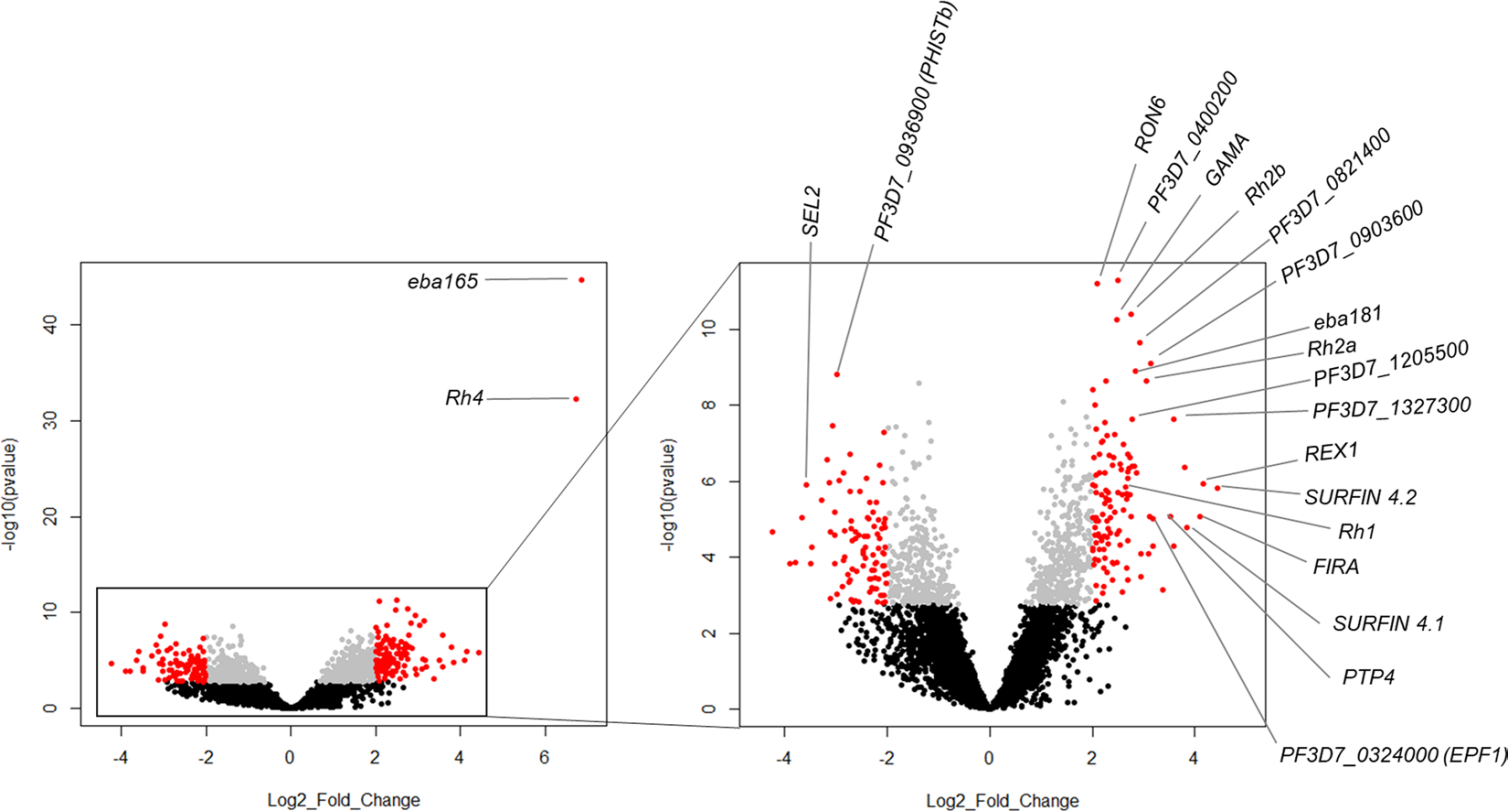
Genes differentially expressed between static and suspended W2mef. Volcano plot showing the extent and significance of differentially expressed genes between suspended and baseline cultures of W2mef. Insert is a zoom in of gene expression excluding the extremely significant genes *Rh4* and *eba165*. Grey: Benjamini-Hochberg adjusted p value < 0.01, red: absolute log2 fold change >2. Genes with symbols have no known function. Black: Benjamini-Hochberg adjusted P value > 0.01 (considered not significant).

To investigate the molecular mechanisms associated with the switching of parasites when cultured in continuous suspension, we first examined differential gene expression in W2mef SP relative to W2mef BL cultures. Focusing on genes with log2 fold change >2 (more than four-fold difference) and adjusted *P* value < 0.01, we identified 212 genes to be differentially expressed, of which 119 and 93 had higher and lower transcript levels, respectively, in W2mef SP relative to W2mef BL (Fig. 2 and Supplementary Table S6). These differentially expressed genes are widely distributed in all chromosomes of the *P. falciparum* genome (Supplementary Fig. S4). We further compared individual SP replicates to the pooled BL samples and observed a similar trend in differential gene expression among replicates as was observed in the bulk analysis (Supplementary Fig. S5). Despite replicate 3 showing a generally reduced fold difference, the topmost genes had expression patterns similar to the other replicates (Supplementary Fig. S5). Removal of this replicate did not alter the results for the initial analysis.

Gene ontology analysis of the most significantly differentially expressed genes showed enrichment for a number of terms (*P* < 0.01; Supplementary Table S7), with heparin binding, sulfur compound binding and glycosaminoglycan binding representing the most enriched terms (*P* < 0.0001 for all). Among the most significantly differentially expressed genes in our dataset were those involved in invasion. These included *eba165*, *eba181*, *Rh1*, *Rh2a*, *Rh2b*, *Rh4*, rhoptry neck protein 6 (RON6) and GPI-anchored micronemal antigen (GAMA) (Fig. 2 and Supplementary Table S6). Additionally, a large number of virulence associated genes, including members of the surface-associated interspersed protein (SURFIN) family, exported protein family 1 (EPF1), ring exported protein 1 (REX1) and interspersed repeat antigen (FIRA); all of which are exported into host erythrocytes, were significantly expressed at higher levels in SP parasites relative to BL (Fig. 2). Other differentially expressed genes included those encoding erythrocyte membrane protein 1 trafficking proteins (PTP4 and PTP5), ring-infected erythrocyte surface antigen (RESA2 and RESA3), bromodomain protein 2 (BDP2), members of the ApiAP2 transcription factors including AP2-G, AP2-O, chromatin modifying proteins such as lysine-specific histone demethylase (LSD2), and proteins of unknown functions (Supplementary Table S6).

**Figure 3:**
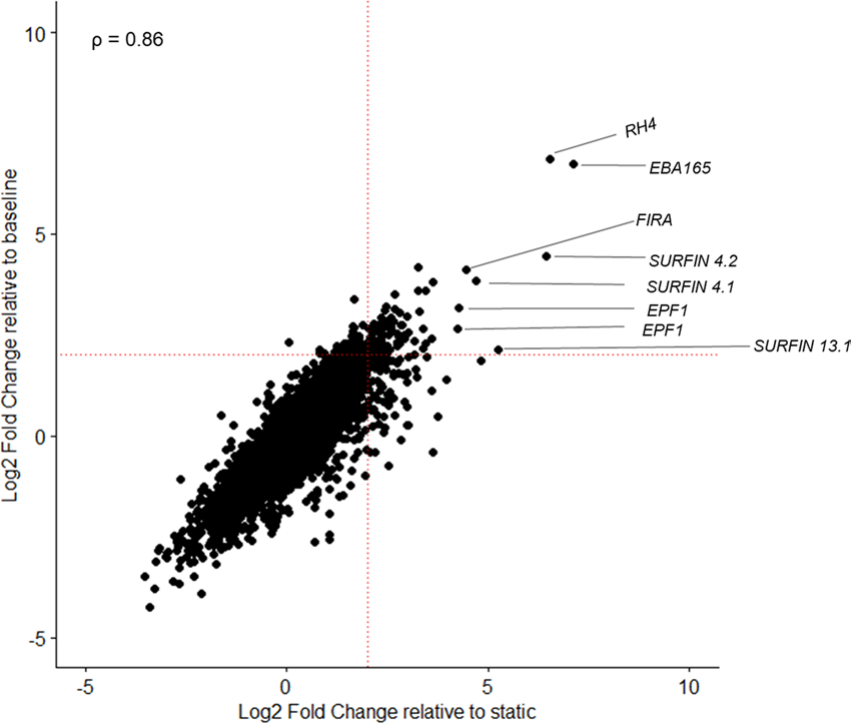
Baseline and static cultures have similar gene expression patterns in comparison to suspended W2mef. Differential expression analyses were conducted for baseline vs suspended (baseline; vertical axis) and then static vs suspended (static; horizontal axis). A spearman rank correlation plot comparing the differential gene expression between baseline and suspended culture (vertical axis) as against static and suspended cultures (horizontal axis) was then constructed using the log2 fold change values from each comparison. ρ is the coefficient of correlation. Red dotted lines indicate the significance threshold which was set at 4-fold increase in gene expression.

To further test these observations, we performed differential expression analyses between the whole transcriptome of static (W2mef ST) and suspended W2mef (W2mef SP) grown for the same length of time, and compared the expression profile to that observed for BL vs SP analysis. The results show concordance in differential expression between SP cultures and either the respective BL or ST cultures, with overall Spearman rank correlation coefficient of 0.86 (Fig. 3). A closer look at the differentially expressed genes show similar patterns, with many invasion-related and exported protein genes being more highly expressed in SP cultures relative to their ST counterparts. Furthermore, we tested the robustness of this approach by pooling all static samples (ST + BL) and compared them to SP samples in a differential expression analysis. A correlation plot of the SP vs either baseline only or pooled static samples gave a strong positive linear correlation, with a correlation coefficient of 0.99 (Supplementary Fig. S6).

**Figure 4:**
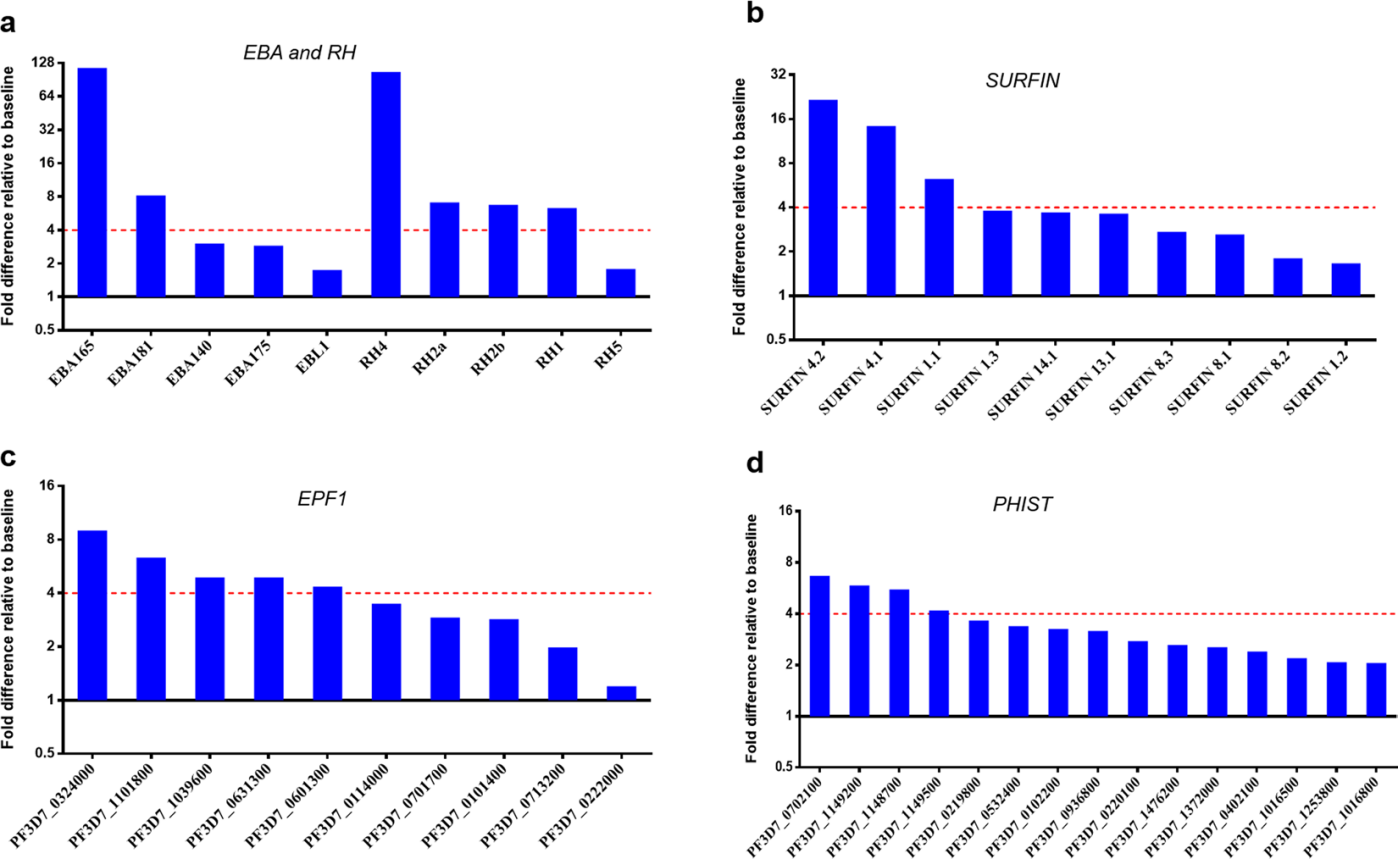
Multi-gene families are differentially expressed between static and suspended W2mef. Differential expression analyses conducted between baseline and suspended W2mef showed increased expressed of specific gene families across the genome, including (**a**) the *eba* and *Rh* gene families, (**b**) *SURFIN* genes, (**c**) exported protein family 1 (*EPF1*) genes and *PHIST* genes. Red dotted lines indicate the significance threshold which was set at 4-fold increase in gene expression.

Focusing on gene families, with the exception of *Rh5*, all the *Rh* genes previously implicated in invasion (*Rh1*, *Rh2a*, *Rh2b* and *Rh4*) were significantly upregulated in SP parasites relative to BL (Fig. 4a and Supplementary Fig. S7). Among the *eba* genes, *eba165* and *eba181* showed significantly higher expression in SP parasites (the pseudogene *eba165* is co-regulated with *Rh4*^39)^, while the others showed a trend in the same direction although at insignificant levels (Fig. 4a). Identification of large numbers of highly expressed exported protein genes prompted us to take a closer look at the transcriptome-wide pattern of some exported protein family members. We observed higher expression levels for all 10 members of the SURFIN family in SP parasites relative to BL, with 8 members having more than two-fold increased expression (Fig. 4b and Supplementary Fig. S7). The genes encoding SURFIN4.1 and SURFIN4.2 which have been localized to merozoites^40,41^, had the largest change in expression levels within this gene family. Interestingly, all 10 genes belonging to the Maurer’s cleft associated exported protein family 1 (EPF1)^42^ also had higher transcript levels in SP parasites compared to BL cultures (Fig. 4c). Likewise, fifteen genes belonging to the *Plasmodium* Helical Interspersed Sub-Telomeric (PHIST) family, had more than two-fold increased expression in SP parasites (Fig. 4d). The PHIST protein genes included the ring-infected erythrocyte surface antigen (RESA3) and PF3D7_0402100; both of which have previously been shown to be essential for parasite survival^43^. Also included in the differentially expressed genes were those coding for transcription factors, with notable ones being AP2-G and other members of the ApiAP2 gene family, as well as bromodomain protein 2 among others (Supplementary Fig. S7 and Supplementary Table S6).

### Epigenetically regulated genes are differentially expressed between moving suspension and static cultures

Previous data have shown that the switched suspended culture phenotype is reversible, and that expression of *Rh4* and *eba165* is epigenetically controlled^17,39,44,45^. Therefore, we sought to find out the extent of expression of epigenetically controlled genes among the larger number of differentially expressed genes here. Heterochromatin protein 1 (HP1) occupancy at the promoter region is a marker for epigenetic gene silencing^45,46^, and can be used as a proxy for the identification of epigenetically regulated genes. Cross-referencing our data with a previously compiled list of HP1-associated genes^47^, we identified 111 genes in our data to have HP1 occupancy. Of these, 16 (14.4%) were significantly differentially expressed in SP parasites (Supplementary Table S8), indicating a high representation of epigenetically controlled genes (Odds Ratio 4.1, *P* = 1.18 × 10^−5^), with 14 (87.5%) of these genes expressed at higher levels in SP parasites.

A recent study identified a large proportion of *P. falciparum* genes to be required for parasite survival^48^. We thus cross-referenced our list of differentially expressed genes with those designated as essential or dispensable. A total of 110 (3.5%) of the 3124 essential genes were differentially expressed in W2mef SP relative to W2mef BL, whereas 102 (5%) of the 2042 dispensable genes were differentially expressed. This shows that a significantly lower than random proportion of essential genes were differentially expressed (Odds Ratio 0.65, *P = *2.98 × 10^−3^).

### Genes differentially expressed in suspended Dd2 relative to baseline

**Figure 5:**
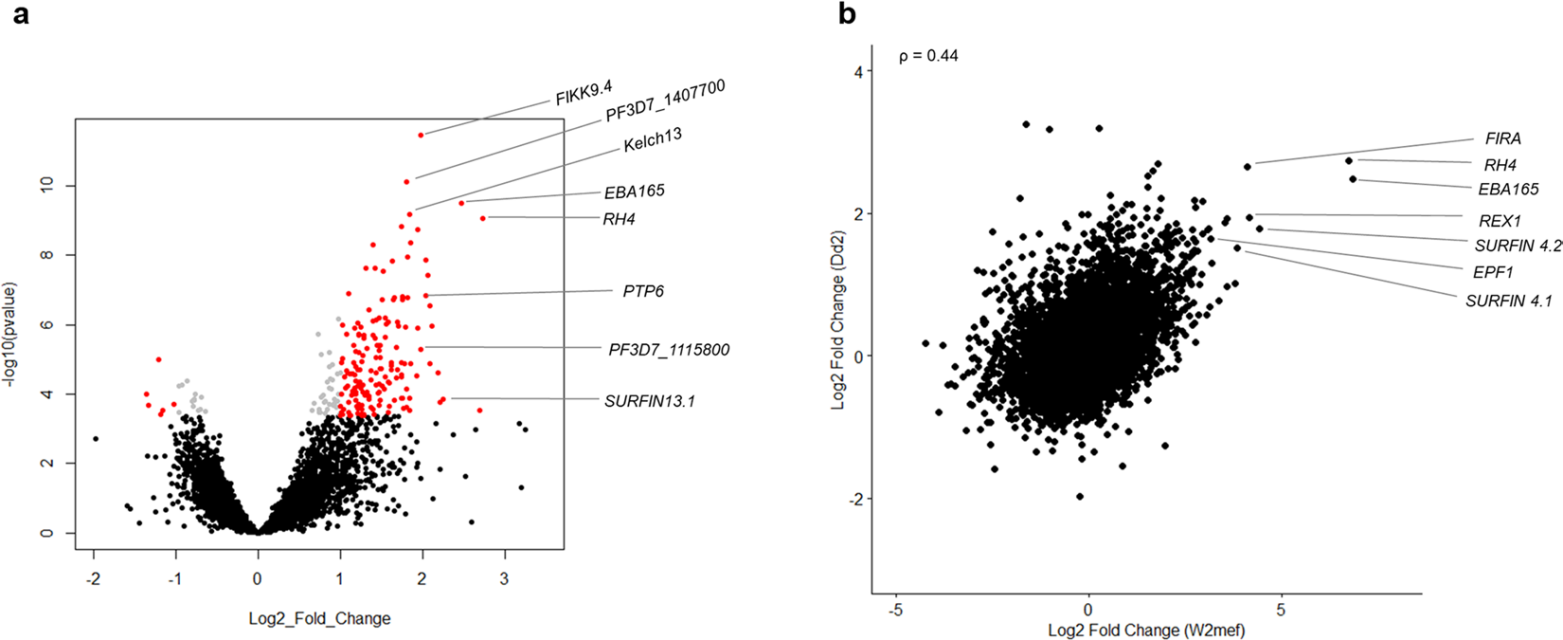
Pattern of differential gene expression is similar between Dd2 and W2mef. (**a**) Differential gene expression analyses between baseline and suspended Dd2 shows increased expression of *Rh4*, *eba165* in addition to other genes to be highly expressed in suspended Dd2. Grey: Benjamini-Hochberg adjusted *P* value < 0.01, red: absolute log2 fold change >1. Genes with symbols have no known function. Black: Benjamini-Hochberg adjusted *P* value > 0.01 (considered not significant). (**b**) A Spearman rank correlation plot of differential gene expression between suspended vs baseline W2mef (horizontal axis) and suspended vs baseline of Dd2 (vertical axis) shows similarities in the expression pattern between suspended cultures of the two strains. *ρ* is the coefficient of correlation.

Having identified genes that are significantly differentially expressed between SP and BL cultures of W2mef, we performed similar differential expression analyses for the Dd2 clone. Generally, the range of fold change values observed for differential expression between Dd2 SP and Dd2 BL were lower (−1.98 to 3.25) compared to those in W2mef (−4.25 to 6.85) (Supplementary Fig. S8), potentially because Dd2 was cultured for a shorter time (6 rather than 8 weeks) or a possible biological difference which might exist between the two apparently isogenic strains when grown in suspension conditions. Using a cutoff of 4-fold difference between groups and adjusted *P* value < 0.01, twelve genes were identified to be differentially expressed between Dd2 SP and Dd2 BL, all of which were more highly expressed in Dd2 SP (Supplementary Fig. S9). A less stringent cutoff of more than 2-fold difference identified 165 genes to be significantly differentially expressed, with 159 of these expressed at higher levels in SP parasites (Fig. 5a and Supplementary Table S9). The pattern of gene expression was similar to that of W2mef, with *Rh4* and *eba165* among the most highly differentially expressed genes. Additionally, a number of exported protein genes as well as functionally unknown ones were more highly expressed in Dd2 SP relative to Dd2 BL. A comparison of differential gene expression in W2mef with Dd2 showed a significant positive correlation (Fig. 5b), with the directionality of expression of most of the highly significantly expressed genes in W2mef remaining similar in Dd2 (Supplementary Table S2 and Supplementary Fig. S10). Similar to W2mef, majority of the individual biological replicates showed concordance with the results from the pooled analyses (Supplementary Fig. S11). Analysis of the genes differentially expressed between Dd2 SP and Dd2 BL for enriched gene ontology terms (*P* < 0.01) identified protein kinase activity and translocation of peptides or proteins into host cell cytoplasm to be among the most enriched terms (Supplementary Table S10). A comparison of the levels of differential expression of the *eba* and *rh* gene between ST and SP parasites in the current RNA sequencing study and our previous RT-qPCR analysis shows a strong positive correlation of results from the two studies (Supplementary Fig. 12).

## Discussion

The specific roles of parasite ligands in erythrocyte invasion phenotype variation are not well-understood and appear to differ between parasite strains^16,18,26,34^. These previous studies have shown that invasion phenotype switching has mainly been through the differential expression of members of the *Rh* or *eba* gene families. We recently demonstrated that the phenotypic switching in *P. falciparum* W2mef and Dd2 clones could be achieved through simply culturing them in moving suspension rather than static conditions^17^. In this report, we have investigated this phenomenon further using whole transcriptome analyses. We show significantly increased expression of four *Rh* and two *eba* genes in SP parasites relative to BL controls, all of which have been implicated in previous invasion phenotype switching studies, except *eba181*^18,26–28,34,49,50^. The mechanistic relevance of the differential expression of multiple invasion-related genes in SP parasites is not yet known, but it is notable that the encoded proteins possess erythrocyte binding properties and have been shown to function during early interactions leading to invasion^13,14,51^. In contrast, the invasion-related genes *ama1* and *Rh5* which function during the later stages of invasion^13^ were not significantly differentially expressed in SP parasites, suggesting that moving suspension culture may possibly select for parasites with stronger interactions to fasten onto the erythrocyte surface prior to entry.

The expression of *Rh2a* and *Rh2b* are of interest, as a recent forward genetic screen showed the *Rh2* locus to be associated with invasion phenotype switch, with higher expression of these ligands leading to increased sensitivity to chymotrypsin treatment^28^. Our previous phenotypic data showed that switched SP parasites were more sensitive to chymotrypsin treatment compared to ST parasites^17^, thus the increased expression of these ligand genes in the switched SP parasites here suggests a possible role of *Rh2a* and *Rh2b* in the phenotypic switch. Increased expression of *Rh2b* and *Rh4* correlates with sialic acid-independent invasion by clinical isolates of *P. falciparum*^52^, suggesting that the two ligands may function cooperatively.

Host erythrocyte remodeling by exported parasite proteins leading to increased adherence and resistance to stress is a prominent virulent property of *P. falciparum*^40,43,53–57^. Increased rigidity and adhesion of infected erythrocytes enhance sequestration through cytoadherence and rosetting, both of which are associated with disease severity^58^. A substantial number of exported proteins were highly expressed in suspended parasites, prominent ones being the SURFINs and EPF1. Both SURFIN4.1 and SURFIN4.2 have been localized to the parasitophorous vacuole and merozoite surface, with SURFIN4.2 suggested to have a role in merozoite invasion^40,41,59^. Available data show that SURFIN4.2 forms a complex with glutamate-rich protein (GLURP) and RON4 in both schizonts and free merozoite, with anti-SURFIN4.2 antibodies partially inhibiting erythrocyte invasion^59^. Additionally, SURFIN4.2 is exported to the erythrocyte surface^40^, with deletion of its gene resulting in reduced erythrocyte membrane rigidity^43^. SURFIN4.1, though annotated as a pseudogene, has been shown to be expressed as a functional protein, with its knockdown resulting in impaired merozoite formation during schizogony^60^. The EPF1 family are Maurer’s cleft associated proteins whose reduced expression results in deficient merozoite release^42^. Efficient merozoite release increases the chances of successful invasion and thus higher parasite growth, which is a hallmark of parasites grown under flow conditions^17,61–63^.

The PHIST proteins are known to be exported to various locations including both the host cell periphery and cytosol, as well as the parasite’s parasitophorous vacuole, Maurer’s cleft and merozoite surface^64–67^. They function by interacting with host erythrocyte cytoskeletal components^64,65,68,69^ as well as parasite-specific proteins such as PfEMP1, skeleton binding protein 1 (SBP1), and knob associated histidine rich protein (KAHRP)^66,70,71^, ultimately leading to cytoadherension and resistance to stress^68,72^. PHIST proteins such as members of the PFEMP1 trafficking protein (PTP) contribute to cytoadherence by mediating the successful trafficking of PfEMP1 from the Maurer’s cleft to the host cell surface^43^. Additionally, PTP2 mediate cell-cell communication by trafficking exosome-like vesicles between infected erythrocytes, a phenomenon which modulates host immune response and increase gametocytogenesis^73–75^. The contents of these vesicles range from parasite DNA, RNA and PfEMP1 proteins^74,76^. Elevated expression of exported protein genes in SP parasites may thus have physiological relevance, as these proteins could potentially affect the rigidity of the erythrocyte membrane to withstand the stress imposed by moving suspension culture conditions, mediate effective communication among parasites, modulate gametocyte production, etc. Another plausible hypothesis for the increased expression of the exported protein genes is to increase the adherent properties of erythrocytes in preparation for stronger binding to possible endothelial molecules in an *in vivo* setting.

Another feature of the current data is the differential expression of genes involved in transcription regulation. We observed increased expression of some members of the ApiAP2 transcription factor family which are involved in the regulation of different processes and stages during parasite development^77–79^. Notably, AP2-G, which regulates gametocyte conversion^78,80–82^ is among the most significantly differentially expressed genes in W2mef SP^79^. This gene is epigenetically controlled, and thus the observation could be a consequence of global upregulation of epigenetic-regulated genes. However, a recent study has shown that AP2-G interacts with AP2-I to drive the expression of invasion related genes, potentially to increase the invasion efficiency of sexually committed merozoites^83^. Given the length of time of culture adaptation and the observation that SP parasites grow better than ST parasites^17^, we postulate that the expressed AP2-G, if functional, is to enhance parasite outgrowth by increasing invasion efficiency. Another AP2 with increased expression in suspended parasites is AP2-EXP which regulates the expression of clonally variant genes^79^. This is unsurprising given the extent of increased expression of exported protein genes in the current dataset.

It is presently unclear how the moving suspension culture condition selects for the phenotype and transcriptional change seen. If parasites have the cellular machinery to sense culture motion, they might alter their phenotype in response, reminiscent of nutrient sensing by the sucrose non-fermenting 1 (SNF1)-related serine/threonine protein kinase KIN^84^. A more general possibility is that a subset of parasites that grow better under such conditions are selected over time, given the length of time it takes for the majority of parasites under suspension culture to acquire the neuraminidase-resistant erythrocyte invasion phenotype. The significant enrichment in expression of epigenetically controlled genes in the SP cultures relative to ST has offered a clue about the possible mechanisms for the changes in gene expression. Elucidating these mechanisms that connect culture agitation with transcriptional changes that mediate phenotypic variation will be the focus of our future studies.

## Materials and Methods

### Parasite culturing and schizonts harvesting

Ethical approval was obtained from the Institutional Review Board of the Noguchi Memorial Institute for Medical Research, University of Ghana, and all methods used in the study were in accordance with the guidelines and regulations provided by the ethical committee. All human erythrocytes used in this study were obtained with written informed consent of the donors. *Plasmodium falciparum* strains W2mef and Dd2 were cultured as previously described^17^. Briefly, Parasites were cultured at 37 °C in RPMI-1640 (Sigma) supplemented with 0.5% Albumax II (Gibco), 20 mg hypoxanthine, 2 g/L sodium bicarbonate (Sigma) and 50 µg/ml gentamicin sulfate (Sigma) using human group O^+^ erythrocytes at 4% hematocrit in a mixed gas environment of 93% nitrogen, 5% CO_2_, and 2% oxygen (Air Liquide, Birmingham, United Kingdom). Cultures were initially grown under static culture conditions for the generation of baseline schizont material for analysis, and the sialic acid-dependent invasion phenotype of both strains was confirmed using a previously described procedure^17^. The true positive discovery rate of differentially expressed genes should increase significantly by using a larger number of biological replicates^85^, with six replicates having previously been recommended for differential expression analyses between different strains of *Plasmodium falciparum*^35^. This study aimed at identifying differentially expressed genes between parasites of the same strain grown under different conditions, thus we projected to study each condition with eight biological replicates. Parasites were maintained as 25 ml cultures at 4% hematocrit, and at about 10% parasitemia with more than 50% rings, the ring stages were selected by treatment with 5% sorbitol^86^. These parasites were cultured to develop to schizont stages, the schizonts were purified by percoll-alanine discontinuous gradient centrifugation^17^, diluted with five-fold more fresh erythrocytes, 10 µM E64 added to prevent egress and cultured for a 6-hour period to allow schizont maturation^35^. Mature schizonts were purified by percoll-alanine, homogenized with 500 µL of TRIzol reagent (Ambion/Life Technologies, Carlsbad, California) and stored at −80 °C until processing for RNA extraction.

After successful harvesting of schizonts from baseline cultures, each of the experimental replicates was divided into two flasks, one kept in a static incubator (static cultures, ST) and the other kept on an orbital shaker rotating at 44 rpm (moving suspended cultures, SP); making 8 ST and 8 SP replicates for each strain. The invasion phenotypes of SP cultures were monitored weekly until invasion efficiency into neuraminidase treated erythrocytes exceeded 50% relative to invasion into untreated erythrocytes, indicating that most parasites had the switched phenotype. All replicates reached this threshold after 6–8 weeks of continuous culturing. Upon switching, schizonts were harvested from all replicate cultures as described above.

### RNA extraction and processing

Frozen TRIzol-homogenized schizonts were thawed at room temperature and total RNA was extracted by the TRIzol method (Invitrogen Corp., Carlsbad, CA). RNA pellets were dissolved in 100 µL RNase-free water. The RNA was purified and DNA removed by DNase I digestion on RNeasy mini columns (Qiagen, Heidelberg, Germany), following which RNA was eluted in 50 µL RNase-free water, and quantified by Qubit High Sensitivity RNA Assay (Thermo Fisher Scientific). For samples containing at least 500 ng RNA, the RNA integrity was checked on an Agilent Bioanalyzer using RNA 6000 Nano reagents and chips (Agilent Genomics, Waldbronn, Germany).

### Transcriptome RNAseq library preparation and Illumina sequencing

Whole mRNA transcriptome library preparation and sequencing was performed using methods as previously described^35^. Briefly, RNA sequencing libraries were prepared with TruSeq Stranded mRNA Library Prep Kit (Illumina) using 500 ng – 1 µg RNA. Quality of libraries were validated on an Agilent Bioanalyzer using DNA 1000 reagents and chips (Agilent Genomics, Waldbronn, Germany) to quantify library sizes and confirm the absence of primer dimers. Libraries were quantified using a KAPA Universal Library Quantification kit (Roche Diagnostics Limited) on a 7500 Fast Real-Time PCR System (Thermo Fisher Scientific) and library concentrations were adjusted for library size. Pooled libraries of 12–15 pM concentrations were sequenced on a MiSeq System (Illumina) using a MiSeq Reagent Kit v3 (Illumina) with 2 × 75 cycles.

### Parasite RNAseq data analyses

Paired-end fastQ files were aligned using HISAT2 (default alignment parameters)^87^, duplicate reads removed with Picard and converted to indexed bam files using SAMtools^88^. Bam files were visualized with Artemis to confirm that majority (>95%) of the reads aligned to exons with minimal overlaps within introns. The bam files were then filtered to exclude reads with MAPQ scores below 60. Reads were counted using the SummarizeOverlaps feature of the GenomicAlignments package^89^ in R, against the *P. falciparum* 3D7 version 3.0 reference genome sequence using an annotation file that had masked out extremely polymorphic gene regions, duplicated genes, and the *var*, *rifin* and *stevor* large subtelomeric gene families so that they were not included in the analysis^35^. Differential expression analysis was conducted with DESeq2^36^ in R. Briefly, DESeqDataSet was constructed from the SummarizedExperiment object and low expressed genes filtered out. To model for batch effect, “batch” was included in the design formula. Fragments Per Kilobase of transcript per Million mapped reads (FPKM) were made from the DESeqDataSet. Results tables were generated from the DESeqDataSet using Wald test and differential expression analyses conducted with the DESeq2 results function. A combination of differential expression (log2 fold change values) and level of significance (Benjamini-Hochberg adjusted *P* values) were used to identify significantly differentially expressed genes between SP parasites and their corresponding BL or ST cultures. Karyotype was constructed with the karyoploteR package^90^ in R. Principal Component Analyses were performed with DESeq2 using default parameters and plotted with ggplot.

## Supplementary information

Supplementary figures

Supplementary Dataset 1

Supplementary Dataset 2

Supplementary Dataset 3

Supplementary Dataset 4

Supplementary Dataset 5

Supplementary Dataset 6

Supplementary Dataset 7

Supplementary Dataset 8

Supplementary Dataset 9

Supplementary Dataset 10

## Data Availability

All RNA sequencing data are available for access at Gene Expression Omnibus (https://www.ncbi.nlm.nih.gov/geo/), accession number: GSE129949.
