# Supplementary material for: Investigating a Plasmodium falciparum erythrocyte invasion phenotype switch at the whole transcriptome level
Source: Sci Rep. 2020 Jan 14;10:245. doi: 10.1038/s41598-019-56386-y (PMC6959351; doi:10.1038/s41598-019-56386-y)
Supplement: Supplementary file 1 — Supplementary figures [file 41598_2019_56386_MOESM1_ESM.docx]

# **Investigating a *Plasmodium falciparum* erythrocyte invasion phenotype switch at the whole transcriptome level**

Prince B. Nyarko^1,2^, Sarah J. Tarr^3^, Yaw Aniweh^1^, Lindsay B. Stewart^3^, David J. Conway^3^ and Gordon A. Awandare^1,2*^.

^1^West African Centre for Cell Biology of Infectious Pathogens, ^2^Department of Biochemistry, Cell and Molecular Biology, University of Ghana, ^3^Department of Pathogen Molecular Biology, London School of Hygiene and Tropical Medicine, London, WC1E 7HT, United Kingdom.

* Correspondence: Gordon A. Awandare, West African Centre for Cell Biology of Infectious Pathogens, Department of Biochemistry, Cell and Molecular Biology, College of Basic and Applied Sciences, Volta Road, University of Ghana. P. O. Box LG 54, Legon, Accra. Email: [gawandare@ug.edu.gh](mailto:gawandare@ug.edu.gh). Telephone: +233 303 933 223, Ext. 7500.

**Supplementary data**

**Supplementary Figure S1**


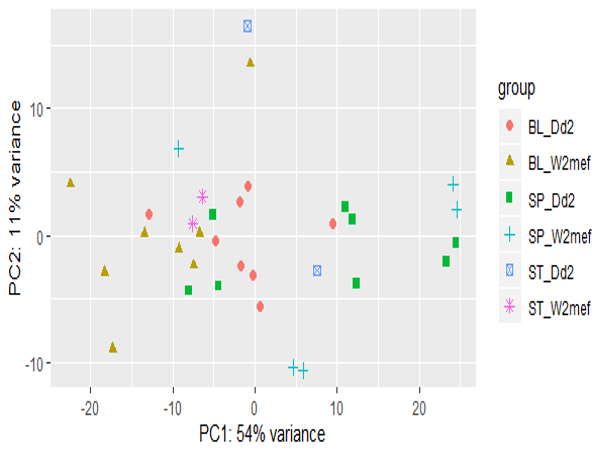


**Supplementary Figure S1: Principal component analysis of all samples analysed**. Samples show no distinct group clustering despite the baseline and static samples marginally separating from some of the suspended samples. BL-baseline, SP-suspended, ST-static.

**Supplementary figure S2**

**
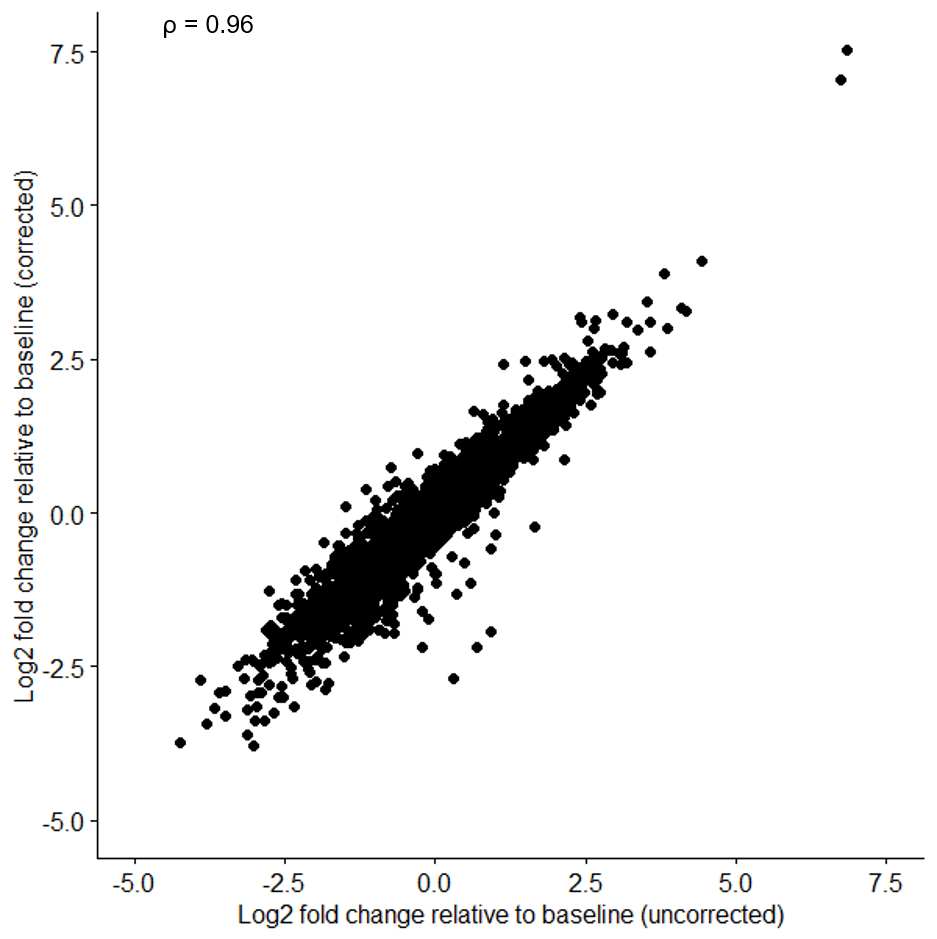
**

**Supplementary Figure S2: Differential expression results is independent of batch effect**. A comparison of gene expression analysis with (vertical axis) or without (horizontal axis) correction for batch effect and outliers shows similar expression patterns.

**Supplementary Figure S3**


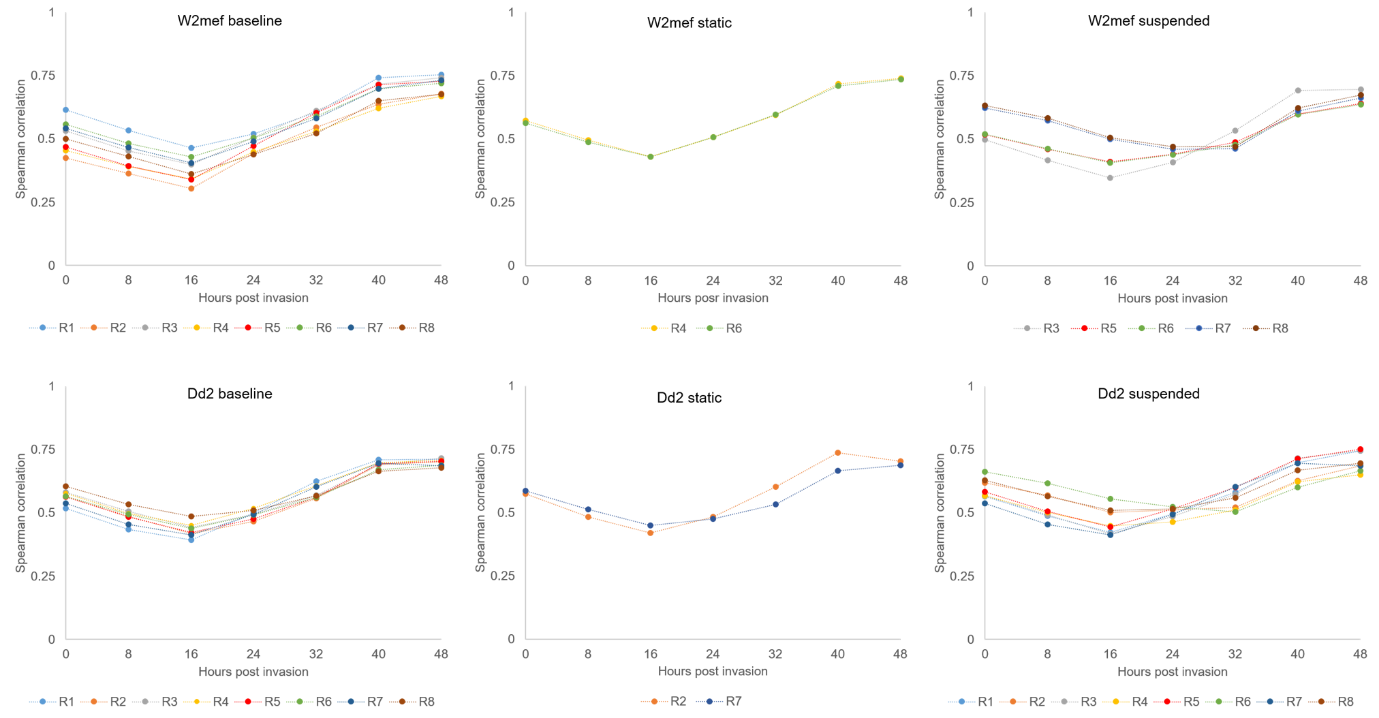


# **Supplementary Figure S3: All samples have schizont-stage specific expression profile**. The FPKM of all samples were compared to the FPKM of data generated by RNA sequencing from a time course expression experiment. All samples in our study show maximum correlation with parasites at 40-48 hours post invasion.

**Supplementary Figure S4**


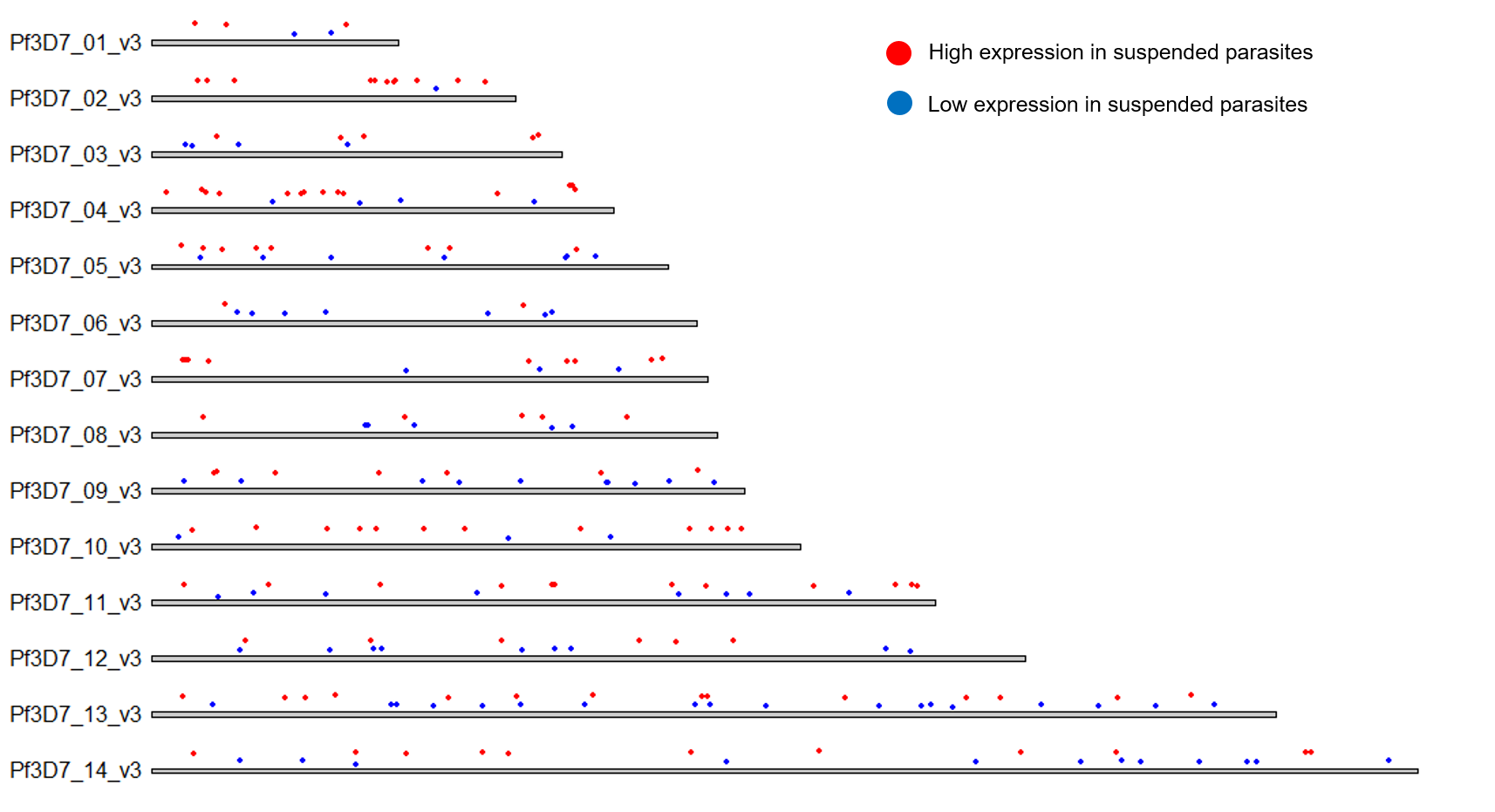


# **Supplementary Figure S4: Differentially expressed genes are distributed across the entire genome**. A karyotype plot of the genomic positions against the log2 fold change of differentially expressed genes between baseline and suspended W2mef (vertical axis not shown). Red - increased expression, blue - decreased expression, in suspended parasites. Karyotype was constructed with the karyoploteR package [81] in R.

**Supplementary Figure S5**


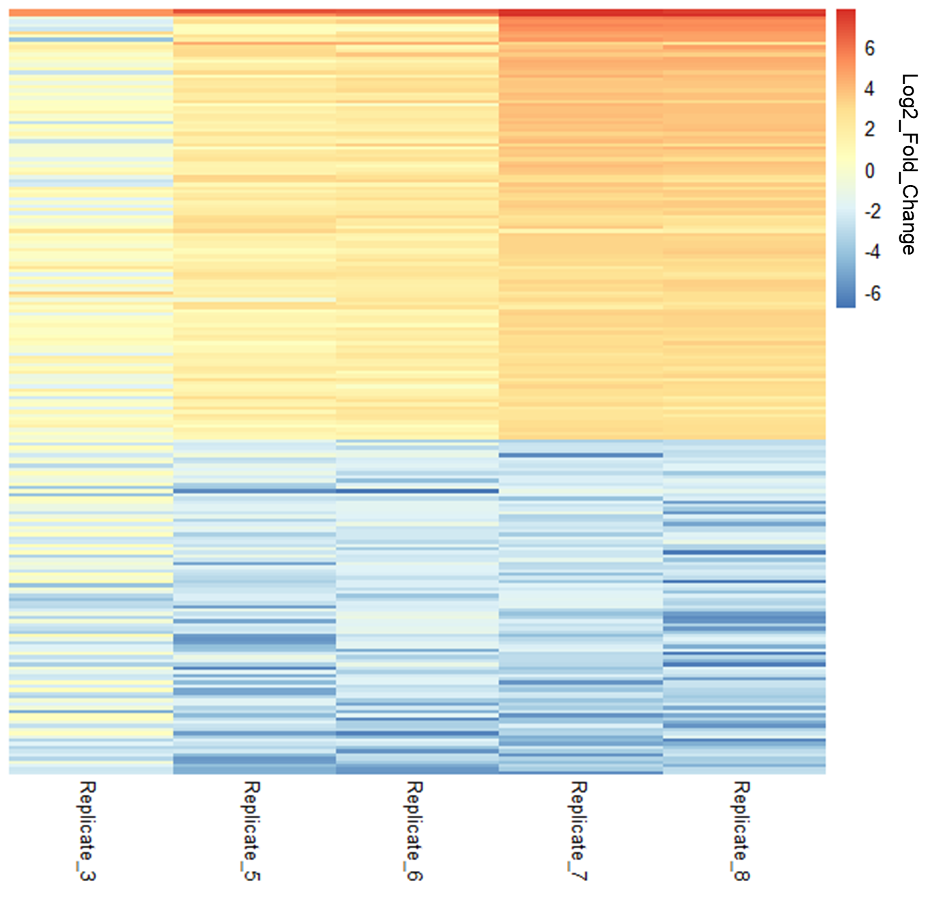


# **Supplementary Figure S5: Expression pattern of significantly differentially expressed genes in individual biological replicates of W2mef**. Individual suspended samples were compared to the pooled baseline samples to determine the replicate-specific expression of all significantly differentially expressed genes in the pooled analyses. Generally, all replicates exhibited similar patterns of gene expression.

**Supplementary Figure S6**


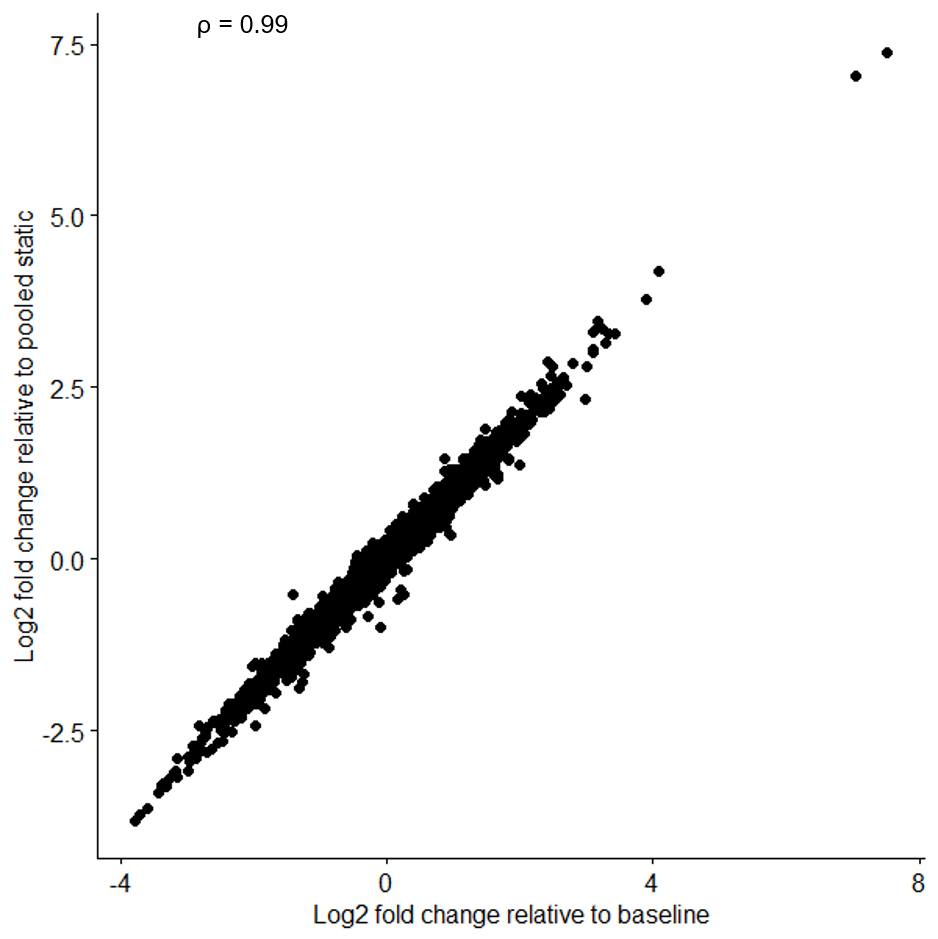


**Supplementary Figure S6: Baseline samples provide adequate representation for all static samples**. A comparison of differential expression data for suspended vs baseline only and suspended vs pooled static samples (baseline + static) shows a near perfect linear correlation.

**Supplementary Figure S7**


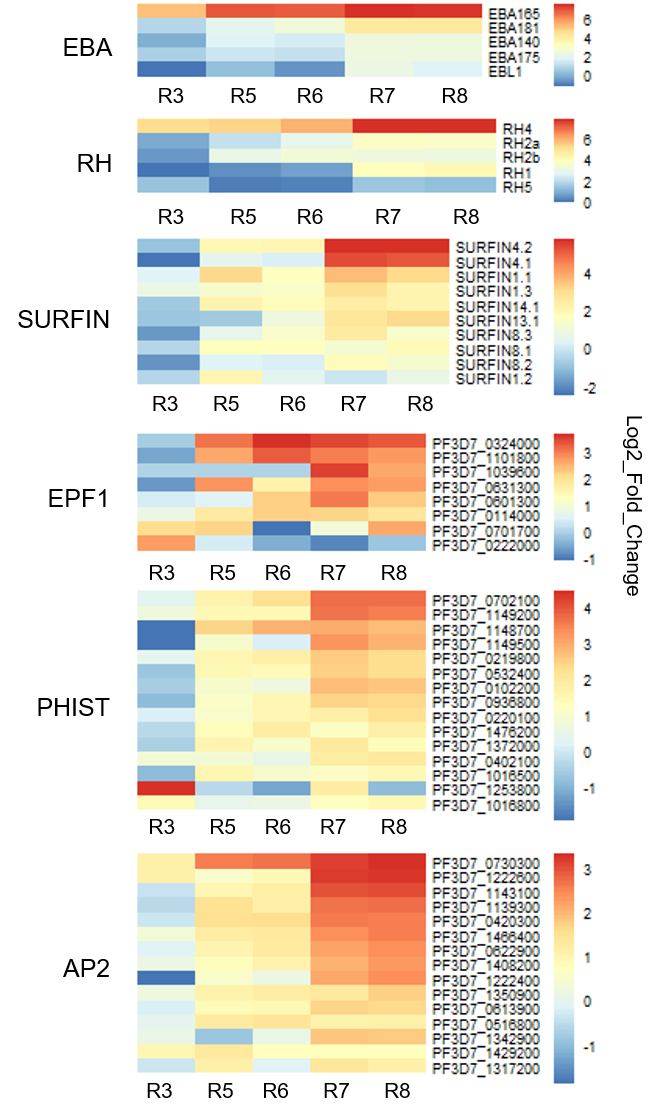


# **Supplementary Figure S7: Expression patterns of highlighted gene families in individual biological replicates of suspended W2mef**. The replicate-specific expression of members of EBA, RH, SURFIN, EPF1, PHIST and AP2 were determined for individual biological replicates of suspended samples by comparing them to the pooled baseline samples (8 replicates) to ascertain per replicate expression variations. The majority of individual replicates exhibited similar patterns of gene expression comparable to the pooled analyses.

**Supplementary Figure S8**


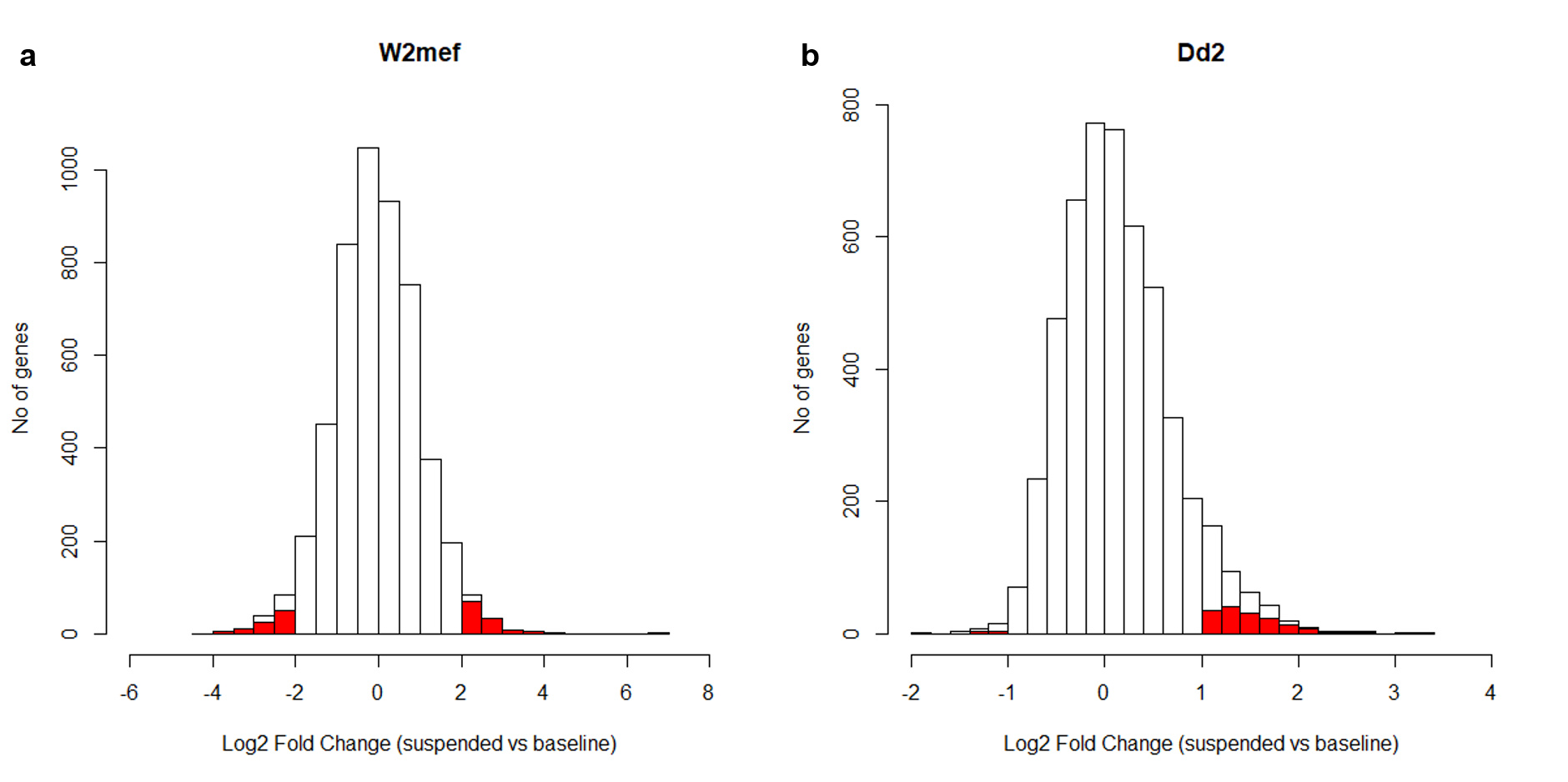


# **Supplementary Figure S8: Fold differences in differential expression are high in W2mef relative to Dd2**. While the log2 fold change values for differential expression between baseline vs suspended W2mef ranged from -4.25 to 6.85 that of baseline vs suspended Dd2 ranged from -1.98 to 3.25. Similarly, the significantly differentially expressed genes between baseline and suspended W2mef had higher fold difference values compared to that of Dd2. Red inserts illustrate genes which satisfy the respective cutoff criteria used to define significance in differential expression in the analyses.

**Supplementary Figure S9**


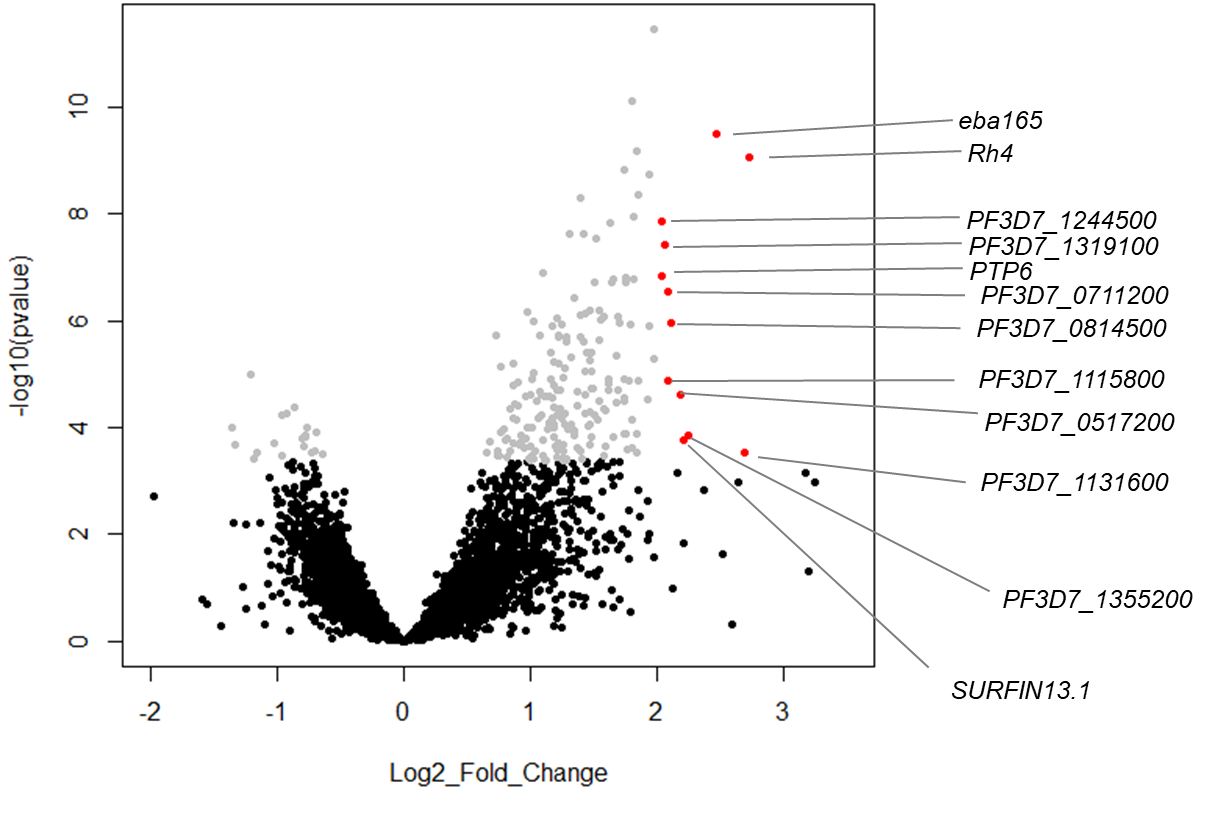


# **Supplementary Figure S9: The most highly significantly differentially expressed genes between baseline and suspended Dd2**. Differentially expression analyses between baseline and suspended Dd2 identifies 12 genes to be significantly differentially expressed with more than 4-fold difference (red). Grey dots are genes with Benjamini-Hochberg adjusted p value < 0.01. Black: Benjamini-Hochberg adjusted p value > 0.01 (considered not significant).

**Supplementary Figure S10**


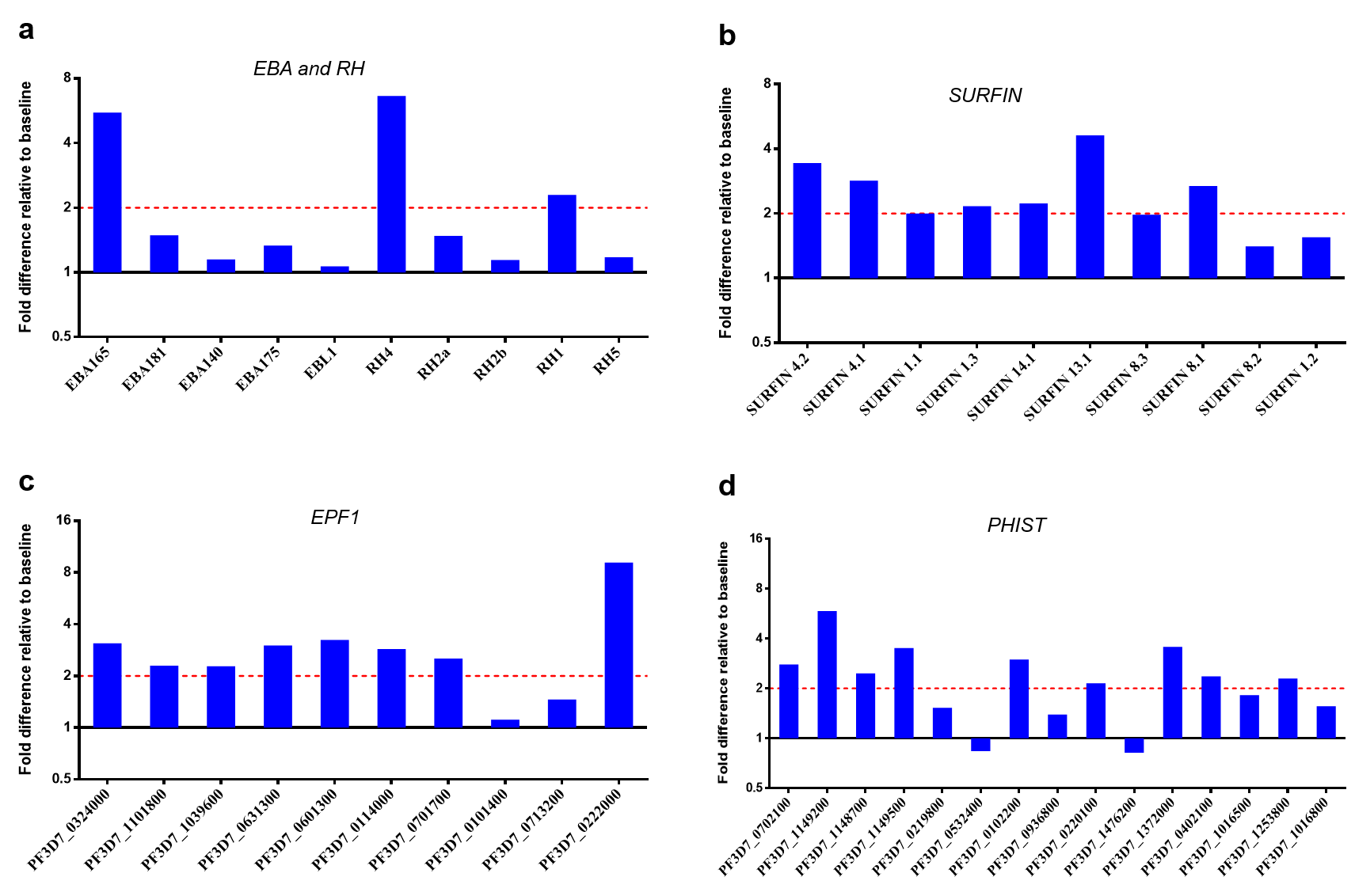


**Supplementary Figure S10: Expression pattern of multi-gene families in suspended Dd2**. The transcriptome-wide expression of (a) *eba* and *Rh*, (b) *SURFIN*, (c) *EPF1* and (d) *PHIST* genes were compared between baseline and suspended Dd2 similar to what was done for W2mef. A general trend of increased expression of the members of gene families was observed, although at a lower level when compared to W2mef (particularly striking for *Rh2a* and *Rh2b*). Dotted lines indicate fold difference considered significant in the analyses.

**Supplementary Figure S11**


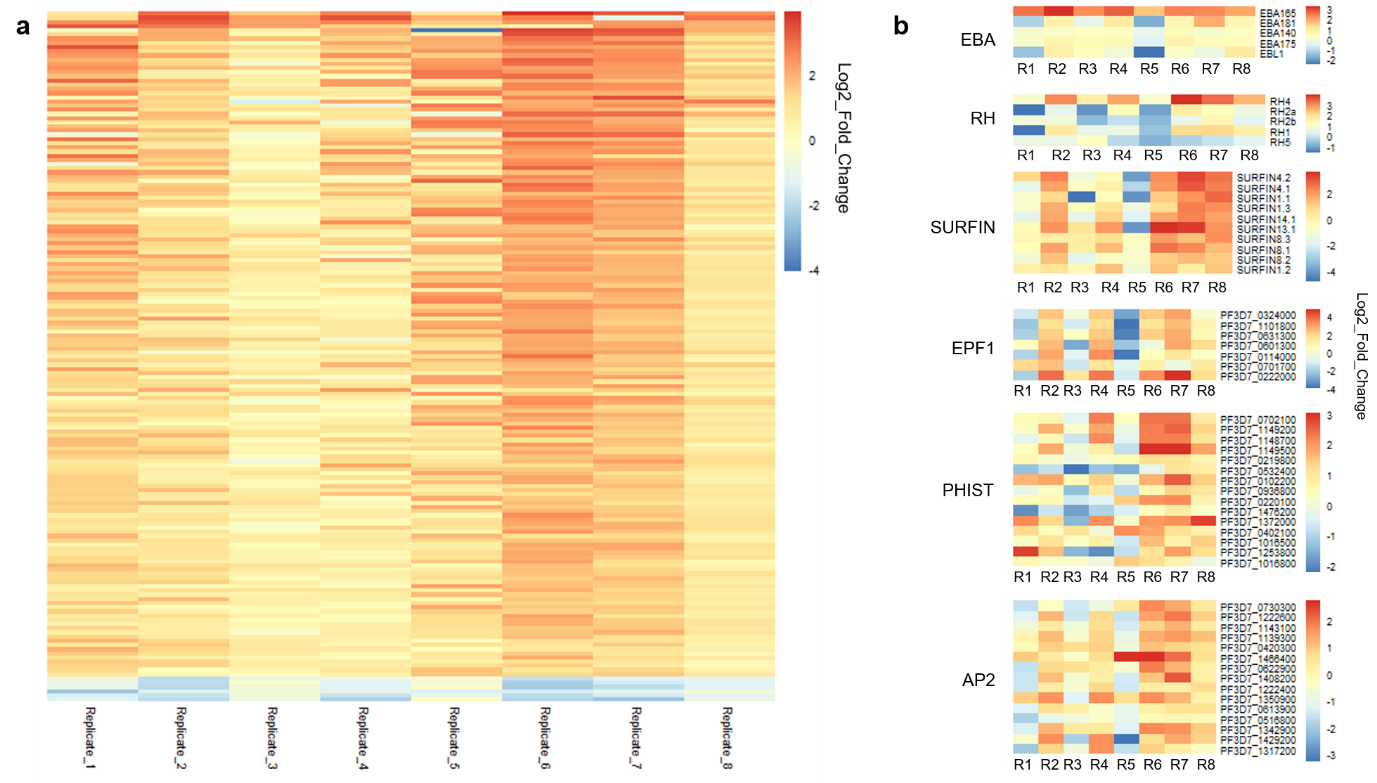


# **Supplementary Figure S11: Expression patterns of highlighted gene families in individual biological replicates of suspended Dd2**. The replicate-specific expression of (a) all the significantly differentially expressed gene and (b) members of EBA, RH, SURFIN, EPF1, PHIST and AP2, were determined for individual biological replicates of suspended samples to identify variations among replicates. Generally, most of the replicates showed similar patterns of gene expression comparable to the pooled samples.

**Supplementary Figure S12**


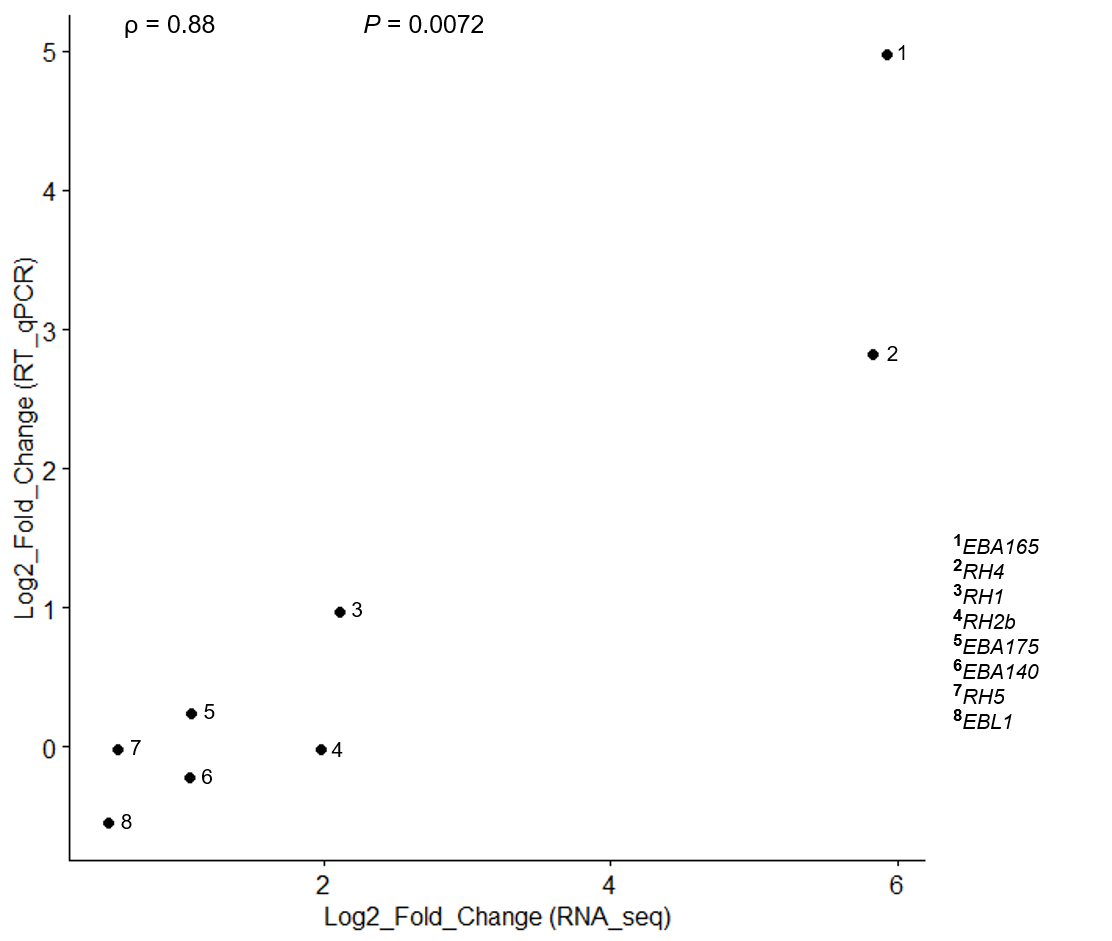


# **Supplementary Figure S12: RNA sequencing correlates positively with RT-qPCR**. Expression of 8 (*eba* and *rh*) genes in the RNA sequencing data were compared to previous data generated from RT-qPCR. Both methods show strong positive correlation of differential gene expression between static and suspended parasites.
